# Protocol and Statistical Analysis Plan for a Multicenter Randomized Trial of Ketamine vs Etomidate for Emergency Tracheal Intubation

**DOI:** 10.1016/j.chstcc.2025.100177

**Published:** 2025-08-13

**Authors:** Stephanie C. DeMasi, Brant Imhoff, Ariel A. Lewis, Kevin P. Seitz, Brian E. Driver, Kevin W. Gibbs, Adit A. Ginde, Stacy A. Trent, Derek W. Russell, Amelia L. Muhs, Matthew E. Prekker, John P. Gaillard, Daniel Resnick-Ault, L. Jane Stewart, Micah R. Whitson, Graham W. W. Van Schaik, Aaron E. Robinson, Jessica A. Palakshappa, Neil R. Aggarwal, Jason C. Brainard, David J. Douin, Carolynn Lyle, Sheetal Gandotra, Aaron J. Lacy, Kristen C. Sherlin, Greta K. Carlson, J. Maycee Cain, Brianne Redman, Carrie Higgins, Cori Withers, Logan L. Beach, Barbara Gould, Jasmine McIntosh, Bradley D. Lloyd, Tiffany L. Israel, Li Wang, Todd W. Rice, Wesley H. Self, Jin H. Han, Jonathan D. Casey, Matthew W. Semler

**Affiliations:** **AFFILIATIONS:** From the Department of Emergency Medicine (S. C. D., G. W. W. V. S., A. J. L., W. H. S., and J. H. H.), the Department of Biostatistics (B. I. and L. W.), the Vanderbilt Institute for Clinical and Translational Research (A. A. L., B. D. L., T. L. I., T. W. R., W. H. S., J. D. C., and M. W. S.), the Division of Allergy, Pulmonary, and Critical Care Medicine (K.P. S., A. L. M., T. W. R., J. D. C., and M. W. S.), the Department of Pharmacy (K. C. S.), Vanderbilt University Medical Center, the Geriatric Research, Education, and Clinical Center (J. H. H.), Tennessee Valley Healthcare System, Nashville, TN; the Department of Emergency Medicine (B. E. D., M. E.P., A. E. R., and G. K. C.), the Division of Pulmonary, Allergy, and Critical Care Medicine (M. E.P. and G. K. C.), Department of Medicine, Hennepin County Medical Center, Minneapolis, MN; the Section of Pulmonary, Critical Care, Allergy and Immunologic Disease (K. W. G., J. A.P., and J. M. C.), Department of Medicine, the Department of Anesthesiology-Critical Care (J.P. G.), the Department of Emergency Medicine (J.P. G. and B. R.), Wake Forest School of Medicine, Winston-Salem, NC; the Department of Emergency Medicine (A. A. G., S. A. T., D. R.-A., and C. W.), the Department of Anesthesiology (L. J. S., J. C. B., and D. J. D.), University of Colorado School of Medicine; the Division of Pulmonary Sciences and Critical Care Medicine (N. R. A. and C. H.), Department of Medicine, University of Colorado Anschutz Medical Center, Aurora; the Department of Emergency Medicine (S. A. T. and C. L.), the Department of Anesthesiology (L. J. S.), Denver Health Medical Center (B. G.), Denver, CO; the Division of Pulmonary, Allergy and Critical Care Medicine (D. W. R., M. R. W., and S. G.), Department of Medicine, the Department of Emergency Medicine (M. R. W. and L. L. B.), University of Alabama at Birmingham, the Pulmonary Section (D. W. R.), Birmingham Veteran’s Affairs Medical Center (J. M.), Birmingham, AL.

**Keywords:** emergency tracheal intubation, etomidate, induction, ketamine

## Abstract

**BACKGROUND:**

Emergency tracheal intubation is a common and high-risk procedure. Ketamine and etomidate are medicines commonly used to induce anesthesia for emergency tracheal intubation, but whether the induction medication used affects patient outcomes is uncertain.

**RESEARCH QUESTION:**

Does the use of ketamine for induction of anesthesia decrease the incidence of death among adults undergoing emergency tracheal intubation compared with the use of etomidate?

**STUDY DESIGN AND METHODS:**

The Randomized Trial of Sedative Choice for Intubation (RSI) is a pragmatic, multicenter, unmasked, parallel-group randomized trial being conducted at 14 sites (6 emergency departments and 8 ICUs) in the United States. The trial compares ketamine vs etomidate for induction of anesthesia among 2,364 critically ill adults undergoing emergency tracheal intubation. The primary outcome is all-cause 28-day in-hospital mortality. The secondary outcome is the incidence of cardiovascular collapse during intubation, a composite of hypotension, receipt of vasopressors, and cardiac arrest.

**RESULTS:**

Enrollment began on April 6, 2022, and is expected to conclude in 2025.

**INTERPRETATION:**

The RSI will provide important data on the effects of ketamine vs etomidate on death and other outcomes for critically ill adults undergoing emergency tracheal intubation. Specifying the protocol and statistical analysis plan before the conclusion of enrollment increases the rigor, reproducibility, and transparency of the trial.

**CLINICAL TRIAL REGISTRATION:**

ClinicalTrials.gov; No.: NCT05277896; URL: www.clinicaltrials.gov

Millions of critically ill adults undergo emergency tracheal intubation every year worldwide,^1^ approximately 30% of whom die before hospital discharge.^2,3^ Nearly all patients undergoing emergency tracheal intubation in an emergency department (ED) or ICU receive a medication to induce anesthesia for the procedure.^2,4^ Whether the medication used to induce anesthesia affects patient outcomes is uncertain.

Etomidate, an imidazole-derived sedative-hypnotic agent that produces anesthesia by acting on g-aminobutyric acid receptors, is the medication most often used to induce anesthesia during emergency tracheal intubation in some EDs and ICUs.^4-6^ Etomidate has been described as an “ideal” medication for induction of anesthesia in critically ill adults because of its rapid onset, short duration of action, and limited effect on BP.^7^ However, etomidate inhibits 11-β-hydroxylase in the adrenal glands, which decreases cortisol production and causes adrenal insufficiency for 24 to 72 hours.^8,9^ Among critically ill adults, many of whom experience critical illness-related corticosteroid insufficiency resulting from sepsis or other causes,^10-12^ etomidate-induced adrenal insufficiency has been hypothesized to be associated with organ dysfunction and death.^5^ Concern that adrenal insufficiency caused by etomidate may decrease survival has led federal regulatory agencies in some countries not to approve etomidate for clinical use.^13^

Ketamine, a dissociative agent that produces anesthesia by acting on N-methyl-D-aspartate receptors, is the medication most often used to induce anesthesia during emergency tracheal intubation in some EDs and ICUs.^14,15^ In contrast to etomidate, ketamine increases cortisol production.^16,17^ However, whereas ketamine generally increases BP by stimulating the release of catecholamines,^18^ it is a direct negative inotrope^19-22^ and has been reported to precipitate rare episodes of severe hypotension^14,23-25^ or even cardiac arrest, particularly among critically ill patients with depleted stores of endogenous catecholamines.^24,26^

Previous small to moderate randomized trials comparing ketamine and etomidate for emergency tracheal intubation have reported conflicting results, with some finding that use of ketamine decreased short-term mortality^24,27^ and others finding no significant differences in outcomes.^28-30^ Currently, whether use of ketamine decreases mortality for critically ill adults undergoing emergency tracheal intubation compared with the use of etomidate remains uncertain.

To address this uncertainty, we designed the Randomized Trial of Sedative Choice for Intubation (RSI), which will compare ketamine vs etomidate for induction of anesthesia among 2,364 adults undergoing emergency tracheal intubation. We hypothesize that use of ketamine will decrease the incidence of death compared with etomidate.

## Study Design and Methods

This article was written in accordance with Standard Protocol Items: Recommendations for Interventional Trials guidelines (Table 1, section 2 of e-Appendix 1).^31^

### Engagement of Patients, Families, and Community Members

Detailed information on engagement with survivors of emergency tracheal intubation, family members, and community members in each phase of the design and conduct of the RSI are summarized in section 3 of e-Appendix 1.

### Study Design

The RSI is a pragmatic, multicenter, unmasked, parallel-group randomized trial comparing the use of ketamine vs etomidate for induction of anesthesia in 2,364 critically ill adults undergoing emergency tracheal intubation in 14 sites (6 EDs and 8 ICUs) in the United States. The primary outcome is all-cause 28-day in-hospital mortality. The trial is being conducted by the Pragmatic Critical Care Research Group (www.pragmaticcriticalcare.org). The trial was registered before initiation of enrollment (ClinicalTrials.gov Identifier: NCT05277896).

### Ethics and Regulatory Approval

The RSI protocol was approved by the institutional review board at Vanderbilt University Medical Center (Identifier: 210500) and the US Food and Drug Administration (Investigational New Drug Identifier: 141424). The study is being conducted with Exception From Informed Consent Requirements for Emergency Research (21 CFR 50.24).^32^ Whenever feasible, patients or their legally authorized representatives are approached by research personnel to provide prospective, written informed consent to participate in the trial. When prospective, written informed consent is infeasible, patients are enrolled under Exception From Informed Consent Requirements for Emergency Research and the patient, the patient’s legally authorized representative, or a family member is notified of enrollment in the trial at the earliest feasible opportunity (additional details in section 4 of e-Appendix 1). Plans for community consultation and public disclosure were approved by the single institutional review board at Vanderbilt University Medical Center. The institutional review board of each participating site provided local context for the community consultation and public disclosure plan and activities.

### Study Population

Critically ill adults undergoing emergency tracheal intubation with sedation in a participating unit are potentially eligible. The trial excludes patients who are pregnant, incarcerated, or younger than 18 years. Complete lists of inclusion and exclusion criteria are provided in Table 2.

### Randomization and Treatment Allocation

Patients are randomized in a 1:1 ratio to ketamine or etomidate in permuted blocks of variable size, stratified by study site. Study group assignments are generated using a computerized randomization sequence, placed in sequentially numbered opaque envelopes, and distributed to enrolling sites. Before opening the envelope, the clinician performing the tracheal intubation procedure (referred to as the operator) determines that the patient meets eligibility criteria. Study group assignment remains concealed to study personnel and clinicians until after the decision has been made to enroll the patient and the envelope is opened. Patients are enrolled and randomized as soon as the operator opens the trial envelope to reveal study group assignment. After randomization, patients, clinicians, and study personnel are not masked to trial group assignment.

### Study Interventions

#### Ketamine Group:

For patients in the ketamine group, clinicians are instructed to administer IV ketamine to induce anesthesia for emergency tracheal intubation. The dose of ketamine is determined by the clinicians based on the clinical condition of the patient. A nomogram on the study group assignment sheet provides clinicians with doses of ketamine (in milligrams) that correspond to a full dose (2.0 mg/kg), an intermediate dose (1.5 mg/kg), and a reduced dose (1.0 mg/kg) for a range of patient weights (section 5 of e-Appendix 1).^33^

#### Etomidate Group:

For patients in the etomidate group, clinicians are instructed to administer IV etomidate to induce anesthesia for emergency tracheal intubation. The dose of etomidate is determined by the clinicians based on the clinical condition of the patient. A nomogram on the study group assignment sheet provides clinicians with doses of etomidate (in milligrams) that correspond to a full dose (0.3 mg/kg), an intermediate dose (0.25 mg/kg), and a reduced dose (0.2 mg/kg) for a range of patient weights (section 6 of e-Appendix 1).

### Cointerventions:

The RSI determines the initial primary medication administered for induction of anesthesia during the emergency tracheal intubation procedure. Subsequent boluses or infusions of sedative medications are determined by clinicians. Other aspects of emergency tracheal intubation (eg, fluid and vasopressor management before intubation, choice of neuromuscular blocking medication) are determined by treating clinicians according to clinical protocols in the study units. Cointerventions that potentially could modify the effect of ketamine or etomidate on patient outcomes (eg, administration of corticosteroids after intubation) are recorded prospectively.

### Data Collection

An observer not directly involved with the intubation procedure collects data in real time for key periprocedural outcomes, including oxygen saturation and systolic BP at the time of induction and lowest oxygen saturation, lowest systolic BP, and administration of vasopressors between induction and 2 minutes after intubation. Observers are either research personnel or trained clinical personnel (eg, physicians or nurses). The accuracy of this method of data collection has been validated previously^34^ and has been used in numerous previous trials of emergency tracheal intubation.^3,4,6^

Immediately after the intubation procedure, the operator completes a paper data collection form to record characteristics of the procedure (eg, Cormack-Lehane grade of glottic view^35^), complications during intubation (eg, cardiac arrest), and the operator’s prior intubating experience. Study personnel at each site review the electronic health record to collect data on baseline characteristics, management before and after laryngoscopy, and in-hospital clinical outcomes such as systolic BP at 24 hours after induction, receipt of vasopressors at 24 hours after induction, ventilator duration, and mortality. Study personnel collect information on mortality occurring after hospital discharge from the electronic health record, public vital statistics records and other public records, and phone calls with patients participating in the long-term outcome assessments at 3 and 12 months (the results of the long-term outcome assessments will be reported separately).

### Primary Outcome

The primary outcome is all-cause 28-day in-hospital mortality, defined as death resulting from any cause occurring between enrollment and 28 days after enrollment with outcome ascertainment ending at hospital discharge.

### Secondary Outcome

The sole secondary outcome is cardiovascular collapse, defined as the occurrence of any of the following between induction and 2 minutes after intubation: (1) systolic BP of < 65 mm Hg, (2) new or increased vasopressor use, (3) cardiac arrest not resulting in death, and (4) cardiac arrest resulting in death.

### Procedural, Clinical, and Safety Outcomes

Table 3 reports the exploratory procedural outcomes, exploratory clinical outcomes, and safety outcomes. Definitions of free-day outcomes are included in section 7 of e-Appendix 1.^4,6^

### Long-Term Outcomes

The definitions, collection, and analysis of survival and functional outcomes (eg, symptoms of posttraumatic stress disorder) at 3 months and 12 months will be prespecified in a separate statistical analysis plan. Because these outcomes will not be available for more than 1 year after completion of enrollment in the trial, these long-term outcomes have been registered separately (ClinicalTrials.org Identifier: NCT06179485)^36^ and will be reported separately from the 28-day outcomes described in this statistical analysis plan.

### Data and Safety Monitoring Board

The composition and responsibilities of the data and safety monitoring board (DSMB) are described in section 8 of e-Appendix 1. The DSMB developed a charter, approved the trial protocol, approved the patient notification forms, and approved the trial monitoring plan before trial initiation of enrollment. The DSMB has the authority to recommend that the trial stop at any point, to request additional data, to request additional interim analyses, or to request modifications to the study protocol.

### Trial Stages

The RSI was funded in 2 stages. First, the single-center feasibility stage of the trial was funded by an award from the National Heart Lung and Blood Institute (Grant: K23HL153584). Second, the multicenter stage of trial was funded by a contract from the Patient-Centered Outcomes Research Institute (PCORI; Identifier: BPS-2022C3-30021). The initial trial protocol specified both stages of the trial and the criteria that had to be met to transition from the single-center stage to the multicenter stage. On August 1, 2023, the DSMB (1) reviewed information on funding for the multicenter stage; (2) reviewed data on enrollment rate, adherence to trial procedures, and completeness and quality of trial data from the single-center stage; and (3) approved transition to the multicenter stage of the trial. Neither the DSMB nor the investigators reviewed any analysis of trial outcomes as part of the transition between stages.

### Interim Analysis

On January 7, 2025, the DSMB reviewed a single interim analysis, prepared by the unmasked study biostatistician at the anticipated halfway point of the trial after enrolment of 1,182 patients. The prespecified stopping boundary for efficacy was a *P* value of < .001 for the difference between trial groups in the primary outcome using a generalized linear mixed-effects model with a random effect for study site and a fixed effect for group assignment (ketamine group vs etomidate group). This conservative Haybittle-Peto boundary will allow the final analysis to be performed using an unchanged level of significance (2-sided *P* value of < .05). After reviewing the interim analysis, the DSMB recommended continuing the RSI without modification.

### Sample Size Estimation

The final sample size for the RSI was calculated at the time of receipt of funding for the multicenter stage of the trial from PCORI (details of the initial sample size calculation are provided in section 9 of e-Appendix 1). Based on prior trials in the same settings,^4,6^ we estimated that the incidence of all-cause, 28-day in-hospital mortality (primary outcome) in the etomidate group would be 30%. The patient and clinician partners recommended that the trial should have adequate statistical power to detect an absolute difference between groups in mortality of approximately 5 percentage points. This difference is more conservative than the median of 8 percentage points (interquartile range, 6-10 percentage points) used as the minimum clinically important difference in the design of prior randomized trials in critical care^37^ and is smaller than the observed difference in mortality between ketamine and etomidate of 6 to 7 percentage points observed in 2 previous trials.^24,28^ We used the bsamsize function in R (R Foundation for Statistical Computing) to calculate that achieving 80% statistical power at a 2-sided α value of .05 to detect a difference in mortality of 5.2 percentage points (30.0% in the etomidate group vs 24.8% in the ketamine group) would require enrollment of 2,308 patients. Anticipating that, like previous Exception from Informed Consent Requirements for Emergency Research trials,^38^ < 3% of participants would discontinue follow-up before ascertainment of the primary outcome, we planned to enroll a total of 2,364 patients (1,182 per group).

### Statistical Analysis Principles

Analyses will be conducted following reproducible research principles using R software. Categorical variables will be presented as number and percentage. Continuous variables will be presented as mean (SD) or median (interquartile range). A 2-sided *P* value of < .05 will define a statistically significant between-group difference in the primary outcome. With a single primary outcome, no adjustment for multiplicity will be made. For analyses of secondary and exploratory outcomes, emphasis will be placed on the magnitude of differences between groups with 95% CIs (which will not be adjusted for multiple comparisons), rather than statistical significance. *P* values will not be presented. Templates for the tables that will be presented in the results manuscript and supplement for baseline, periprocedural, and in-hospital outcome variables are provided in e-Tables 1-3.

### Main Analysis of the Primary Outcome

The primary analysis will be an intention-to-treat comparison of patients randomized to the ketamine group vs patients randomized to the etomidate group regarding the primary outcome of all-cause 28-day in-hospital mortality among all patients in the trial population, except those who withdrew from follow-up before ascertainment of the primary outcome. The primary outcome will be compared between the 2 trials groups using a generalized linear mixed-effects model with a random effect for study site and a fixed effect for group assignment (ketamine group vs etomidate group). The absolute difference in percentages, associated 95% CIs, and a *P* value for the comparison will be presented. A rationale for the primary outcome and its analysis can be found in section 10 of e-Appendix 1.

### Additional Analyses of the Primary Outcome

#### Sensitivity Analyses:

We will assess the robustness of the findings of the primary analysis in a series of sensitivity analyses using different approaches to defining and analyzing the primary outcome. First, we will repeat the primary analysis among all patients in the trial population, with patients who withdrew from follow-up before outcome ascertainment treated as (1) all having experienced the primary outcome or (2) all not having experienced the primary outcome. Second, we will repeat the primary analysis using all-cause, all-location mortality at 28 days (ie, including available information on deaths that occur after hospital discharge). Third, we will repeat the primary analysis using a χ^2^ test, rather than a generalized linear mixed-effects model. Fourth, we will compare survival to day 28 between trial groups using the Kaplan-Meier method. Fifth, we will perform an adjusted analysis comparing the primary outcome between groups using a generalized linear mixed-effects model with a random effect for trial site and fixed effects for trial group assignment and the following prespecified baseline variables: age, sex, race, ethnicity, Area Deprivation Index (a proxy for socioeconomic status and an indicator of neighborhood disadvantage),^39^ rurality,^40^ number of comorbidities, and severity of illness before enrollment as assessed by the Acute Physiology and Chronic Health Evaluation II score.^41^ Continuous variables will be modelled assuming a nonlinear relationship to the outcome using restricted cubic splines with between 3 and 5 knots.

#### Analyses of Heterogeneity of Treatment Effect:

Heterogeneity of treatment effect is nonrandom variation in the magnitude or direction of the effect of a treatment on an outcome across levels of a baseline covariate. We will apply the 3 complementary approaches to analysis of heterogeneity of treatment effect described in the Predictive Approaches to Treatment Effect Heterogeneity Statement: (1) traditional 1-variable-at-a-time subgroup analyses, (2) a risk-modeling approach, and (3) an effect-modeling approach.^42^ Complete details of all 3 approaches are described in section 11 of e-Appendix 1.

Our subgroup analyses will examine whether prespecified baseline variables modify the effect of trial group assignment on the primary outcome using a formal test of statistical interaction in a generalized linear mixed-effects model with the primary outcome as the dependent variable, a random effect for trial site, and independent variables of trial group, the proposed effect modifier, and the interaction between the effect modifier and trial group for each of the following prespecified baseline variables: (1) sepsis or septic shock (yes or no), (2) vasopressor receipt (yes or no), (3) patient location (ED or ICU), (4) adrenal insufficiency or long-term receipt of corticosteroids (yes or no), (5) acute neurologic condition (yes or no), (6) active cardiac condition (yes or no), and (7) baseline risk of the primary outcome (continuous).

### Analysis of the Secondary Outcome

We will perform an unadjusted, intention-to-treat comparison of patients randomized to the ketamine group vs patients randomized to the etomidate group regarding the secondary outcome of cardiovascular collapse. A χ^2^ test will be used to generate between-group differences and the associated 95% CIs.

### Analyses of Additional Outcomes

We will conduct unadjusted, intention-to-treat analyses comparing patients randomized to the ketamine group vs patients randomized to the etomidate group regarding each prespecified exploratory procedural outcome, exploratory clinical outcome, and safety outcome. Between-group differences and the associated 95% CIs will be generated using a χ^2^ test for categorical outcomes and a Wilcoxon rank-sum test for continuous outcomes.

### Handling of Missing Data

We anticipate that no data on the primary outcome will be missing except for patients who withdrew from participation before the collection of the primary outcome. When data are missing for an outcome, we will perform complete-case analysis, excluding patients whose data for the analyzed outcome are missing. In adjusted analyses, missing data for covariates will be imputed using multiple imputations. All variables that included in prespecified statistical models also will be included in the imputation model. This allows for the relationships held between the variables of interest to be as similar as possible with and without imputation. The specific method of multiple imputation used in adjusted analysis uses bootstrapping and predictive mean matching. Samples of nonmissing data are bootstrapped, then fit to an additive regression model to predict missing data. Imputation will be performed using the R statistical software’s aregImpute function from the Hmisc package.

## Discussion

This article reports the rationale, design, and analysis plan for the RSI, a 2,364-patient randomized trial of ketamine vs etomidate for induction of anesthesia among critically ill adults undergoing emergency tracheal intubation. Several elements of the trial design warrant discussion.

The RSI was designed to compare ketamine vs etomidate and does not examine other medications capable of inducing anesthesia for emergency tracheal intubation, such as propofol or benzodiazepines. These comparators were selected for several reasons. First, ketamine and etomidate are the medications most often used for emergency tracheal intubation in many EDs^14^ and ICUs,^5^ particularly in the United States.^3,43-45^ Second, ketamine and etomidate are the only medications consistently used for emergency tracheal intubation in clinical care at the 14 sites participating in the trial.^4,6,15^ Third, although propofol and benzodiazepines are used more commonly by some clinical specialties (eg, anesthesiologists)^46,47^ and in some clinical settings (eg, some ICUs outside of the United States),^2^ observational studies consistently have reported these medications to be associated with higher rates of hypotension during intubation, particularly for patients with preexisting hemodynamic instability.^2,48^ We anticipate that approximately 1 in 4 patients in the RSI will be receiving vasopressors before enrollment. Thus, we designed the trial to compare 2 medications with a hemodynamic profile that permits their use for nearly all critically ill adults, including those with hypotension or shock before intubation.

In the RSI, clinicians determine what dose of the assigned medication each patient receives based on the clinical condition of the patient using a dosing nomogram provided by the trial. The nomogram specifies 2 mg/kg of ketamine and 0.3 mg/kg of etomidate as a full dose. These are the doses specified in the US Food and Drug Administration labeling information for the induction of anesthesia.^33,49^ These also are the doses used in 2 of the largest previous randomized trials comparing ketamine vs etomidate among critically ill adults.^24,28^ For patients who clinicians determine require a lower dose, the nomogram also provides information on an intermediate dose (1.5 mg/kg of ketamine or 0.25 mg/kg of etomidate) and a reduced dose (1.0 mg/kg of ketamine or 0.2 mg/kg of etomidate) across a range of patient weights.

The design of the RSI differs from that of previous trials comparing ketamine vs etomidate in several respects. First, the planned sample size of 2,364 patients is almost 3 times as large as any prior trial and will permit more precise estimates of treatment effect.^24,28,29,50^ Second, unlike prior trials that excluded patients intubated in the ED,^24^ that excluded patients intubated in the ICU,^29^ or that enrolled only patients with sepsis,^50^ the RSI includes a broad range of patients undergoing emergency tracheal intubation in an ED or ICU, which increases generalizability. The only diagnosis excluded from the RSI is a primary diagnosis of trauma before seeking treatment in the ED. Because some prior data suggest that ketamine might increase intracranial pressure^51^ and the results of 1 prior trial suggested a potential difference in treatment effect between patients with and without trauma,^28^ we determined that the effect of ketamine vs etomidate on outcomes for patients with trauma was best evaluated in a separate trial. Third, unlike prior trials that did not collect procedural outcomes^28^ or long-term patient-centered outcomes,^24,28,29,50^ the RSI collects physiologic outcomes during the procedure (eg, cardiovascular collapse during intubation), short-term patient-centered outcomes (eg, death by day 28), and long-term patient-centered outcomes (eg, survival and symptoms of posttraumatic stress disorder at 12 months).

## Interpretation

The RSI will provide important evidence regarding the effect of ketamine vs etomidate on death and other clinical outcomes among critically ill adults undergoing emergency tracheal intubation in an ED or ICU. To aid in the transparency and interpretation of trial results, this article detailing the protocol and statistical analysis plan for the RSI has been finalized before the conclusion of patient enrollment.

## Supplementary Material

1

**Additional information:** The e-Appendix and e-Tables are available online under “Supplemental Data.”

## Figures and Tables

**TABLE 1]:** Schedule of Enrollment, Interventions, and Assessments in the RSI

Time Point	Eligibility Screening: Decision to Perform Tracheal Intubation	Randomization and Allocation: Before Tracheal Intubation	During the Procedure				Final Outcome Assessment at 28 d
			Induction	Tracheal Intubation	0-2 min After Tracheal Intubation	0-24 h After Tracheal Intubation	
Enrollment							
Eligibility screen	X						
Enrollment		X					
Allocation		X					
Interventions							
Ketamine			X				
Etomidate			X				
Screening for contraindications	X	X	X				
Assessments							
Baseline variables	X	X					
Periprocedural variables		X	X	X	X		
Adverse events		X	X	X	X	X	X
Clinical outcomes				X	X	X	X

Information on the collection of outcomes at 3 and 12 months will be reported in a separate statistical analysis plan. Table entries without data mean that data elements were not collected at that time. RSI = Randomized Trial of Sedative Choice During intubation.

**TABLE 2]:** Inclusion and Exclusion Criteria

Inclusion criteria
Patient is critically ill and undergoing emergency tracheal intubation with sedation in an enrolling unit
Planned procedure is orotracheal intubation using a laryngoscope
Planned operator is a clinician expected to perform tracheal intubation in the participating unit routinely
Exclusion criteria
Patient is known to be younger than 18 y
Patient is known to be pregnant
Patient is known to be incarcerated
Patient is known to have an allergy to ketamine or etomidate
Patient is seeking treatment at the emergency department with a primary diagnosis of trauma
Patient or legally authorized representative declines participation during pre-enrollment opt-out conversation or by wearing opt-out bracelet for the RSI
Clinician believes that ketamine is required or contraindicated for the optimal care of the patient
Clinician believes that etomidate is required or contraindicated for the optimal care of the patient
Clinician believes that an induction medication other than ketamine or etomidate is required for the optimal care of the patient
Immediate need for intubation precludes safe performance of study procedures

RSI = Randomized Trial of Sedative Choice for Intubation.

**TABLE 3]:** Study Outcomes

Primary outcome	All-cause 28-d in-hospital mortality
Secondary outcome	Cardiovascular collapse, a composite of any of the following between induction and 2 min after intubation: (1) systolic BP < 65 mm Hg, (2) new or increased vasopressor use, (3) cardiac arrest not resulting in death within 1 h of induction, and (4) cardiac arrest resulting in death within 1 h of induction^a^
Exploratory procedural outcomes	Cormack-Lehane grade of glottic view
	Successful intubation on the first attempt, defined as placement of an endotracheal tube in the trachea with a single insertion of a laryngoscope blade into the mouth and either a single insertion of an endotracheal tube into the mouth or a single insertion of a bougie into the mouth followed by a single insertion of an endotracheal tube over the bougie into the mouth
	Time from induction to successful tracheal intubation
	Lowest oxygen saturation between induction and 2 min after intubation
	Lowest oxygen saturation < 80% between induction and 2 min after intubation
	Highest and lowest systolic BP from induction to 2 min after intubation
	Systolic BP > 180 mm Hg between induction and 2 min after intubation
	Systolic BP < 65 mm Hg between induction and 2 min after intubation
	New or increased vasopressor use between induction and 2 min after intubation
	Cardiac arrest within 2 min of intubation not resulting in death within 1 h of induction^a^
	Cardiac arrest within 2 min of intubation resulting in death within 1 h of induction^a^
Exploratory clinical outcomes	Ventilator-free days to study day 28
	Vasopressor-free days to study day 28
	ICU-free days to study day 28
Safety outcomes	Systolic BP at 24 h after induction
	Receipt of vasopressors at 24 h after induction
	Cardiac arrest receiving CPR between induction and hospital discharge

a Cardiac arrest occurring between induction of anesthesia and 2 min after intubation of the trachea will be considered to be a cardiac arrest that occurred during tracheal intubation. For each case of cardiac arrest occurring during tracheal intubation, we will record whether that cardiac arrest resulted in death. A cardiac arrest occurring during tracheal intubation will be considered to have resulted in death if the patient died before 1 h after induction.

## Take-Home Points

### Study Question:

Does use of ketamine for induction of anesthesia during emergency tracheal intubation decrease the incidence of death compared with use of etomidate?

### Results:

This article describes the protocol and statistical analysis plan for the Randomized Trial of Sedative Choice for Intubation comparing ketamine vs etomidate for induction of anesthesia for emergency tracheal intubation.

### Interpretation:

Prespecifying the full statistical analysis plan before completion of enrollment increases rigor, reproducibility, and transparency of the trial results.

## ABBREVIATIONS:

DSMB: data and safety monitoring board
ED: emergency department
PCORI: Patient-Centered Outcomes Research Institute
RSI: Randomized Trial of Sedative Choice for Intubation
